# Association between renal function and bone mineral density in healthy postmenopausal Chinese women

**DOI:** 10.1186/s12902-019-0476-y

**Published:** 2019-12-26

**Authors:** Shuang Li, Junkun Zhan, Yanjiao Wang, Yi Wang, Jieyu He, Wu Huang, Zhifeng Sheng, Youshuo Liu

**Affiliations:** 1 Department of Geriatrics, Institute of Aging and Geriatrics, The Second Xiangya Hospital, Central South University, 139 Renmin Road, Changsha, Hunan 410011 People’s Republic of China; 2 Department of Metabolism and Endocrinology, The Second Xiangya Hospital, Central South University, Changsha, Hunan 410011 People’s Republic of China

**Keywords:** Renal function, Bone mineral density, Osteoporosis, Postmenopausal women

## Abstract

**Background:**

The relationship between renal function and bone mineral density (BMD) is controversial. The aim of this study was to determine the relationship of renal function with BMD and osteoporosis risk in healthy postmenopausal Chinese women.

**Methods:**

A cross-sectional study was conducted in 776 healthy postmenopausal Chinese women. Dual-energy X-ray absorptiometry was used to measure BMDs. Clinical, demographic, and biochemical data were obtained at the time of image acquisition. Estimated glomerular filtration rate (eGFR) was calculated using a Chronic Kidney Disease Epidemiology Collaboration (CKD-EPI) equation.

**Results:**

Women with eGFR levels of at least 90 ml/min/1.73m^2^ had a lower prevalence of osteoporosis compared with women with decreased eGFR levels (60 ml/min/1.73 m^2^ ≤ eGFR < 90.0 ml/min/1.73 m^2^). BMDs at femoral neck and total hip were significantly lower in the lower eGFR class than the higher class (0.717 ± 0.106 vs 0.744 ± 0.125 g/cm^2^, *P* < 0.01; 0.796 ± 0.116 vs 0.823 ± 0.129 g/cm^2^, *P* < 0.01, respectively). eGFR was positively correlated with BMDs at femoral neck and total hip in unadjusted analysis (*P* < 0.05). After controlling for age, menopausal duration and body mass index (BMI), decreased eGFR was not associated with osteoporosis risk.

**Conclusions:**

After adjustments for age, menopausal duration and BMI, the decline in renal function was not independently associated with osteoporosis risk in healthy postmenopausal Chinese women.

## Background

Renal function declines with age such that close to 95% of women aged ≥75 years have a mild to moderate reduction of renal function [1]. Currently, we are experiencing an unprecedented rise in the number of older adults [2], and consequently, the prevalence of many age-related diseases will increase, including chronic kidney disease (CKD) and osteoporosis [3, 4]. Studies show that patients with renal dysfunction exhibit reduced bone mineral density (BMD) and increased risk of fracture [5–9]. Renal dysfunction influences bone in various ways, such as increasing parathyroid hormone levels, abnormal calcium and phosphate metabolism, and vitamin D deficiency [10, 11]. These effects on mineral homeostasis may be associated with increased bone fragility [12]. However, there are conflicting reports, on whether renal function affects BMD or fracture risk. Elliott et al. [13] showed no association between renal dysfunction and fracture risk. Fujita et al. [14] reported that low renal function is not associated with decreased BMD in community-dwelling elderly men.

Osteoporosis is a serious problem affecting a large number of elderly people. Several studies reported a high prevalence of osteoporosis in postmenopausal women [11, 15, 16]. Due to the increase of proportion of the elderly population, osteoporosis has become a major public health problem around the world [17–19]. It was estimated that the annual number and costs of osteoporotic fractures will reach as high as 5.99 million fractures and $25.43 billion in 2050, respectively [20].

Although other studies have investigated the relationship between renal function and BMD, they are mostly in defined kidney disease [5, 21]. The data about the effect of renal function on BMD and osteoporosis in otherwise healthy Chinese postmenopausal women are scare. Therefore, the aim of the present study was to investigate the relationship of renal function (using the Chronic Kidney Disease Epidemiology Collaboration (CKD-EPI) 2009 equations to calculate estimated glomerular filtration rate (eGFR)) with BMD and osteoporosis risk in healthy Chinese postmenopausal women.

## Methods

### Study population

The design and operation of this study have been described [22]. Briefly, a total of 1096 urban postmenopausal Chinese women were recruited at random. Subjects with any pathological disorders (such as diabetes mellitus, hyperthyroidism, oligomenorrhoea, malabsorption, rheumatoid arthritis or hepatic dysfunction) or subjects using medications (such as glucocorticoids, bisphosphonate, oestrogen, thyroid hormones or statins) known to alter bone metabolism were excluded from our study. Of these, 232 subjects had to be excluded. 15 women were excluded due to lacking of time. Fourteen subjects had to be excluded because of impaired renal function (eGFR < 60 ml/min/1.73m^2^) [23]. After excluding 59 participants with abnormal laboratory test results, 776 qualifying healthy postmenopausal women were selected. All subjects agreed to participate in the study and gave written informed consent. The study was approved by the ethics committee of the Second Xiangya Hospital of Central South University (Changsha, China) and was in compliance with the Helsinki Declaration.

### Clinical measurements

Each participant answered a detailed self-report questionnaire that included name, age, menopausal duration, marital status, educational background, smoking or alcohol intake, physical exercise, list of medications and history of fractures, and underwent a clinical examination. Physical exercise was defined as ≥30 min/day. Weight (kg) and height (cm) were measured and BMI (kg/m^2^) calculated. BMD values were measured by dual-energy X-ray absorptiometry (DXA) fan-beam bone densitometer (Lunar Prodigy Advance, GE Healthcare, Madison, WI, USA) at the total hip, left femoral neck, and lumbar spine (L1–L4). We classified the BMD results as normal BMD: T-score > − 2.5 standard deviation (SD) at lumbar spine, femoral neck and total hip, or osteoporosis: T-score ≤ − 2.5 SD in at least one skeletal site [24, 25].

Blood samples were collected after an overnight fast. Serum calcium (SCa) levels were measured by calcium kit according to the manufacturer’s instructions (MedicalSystem Biotechnology CO., Ltd., Ningbo, China). Serum creatinine (SCr) levels were measured by enzymatic method (Kanto Chemical, Tokyo, Japan). Blood urea nitrogen (BUN) levels were measured by enzymatic method (Abbott, Chicago, USA). eGFRs were estimated using the CKD-EPI 2009 equations [14]. Participants were staged into categories of renal function based on their eGFR alone as follows: (1) normal eGFR (≥ 90.0 ml/min/1.73m^2^), (2) mild decreased eGFR (60 ml/min/1.73m^2^ ≤ eGFR< 90.0 ml/min/1.73m^2^) [23].

### Statistical analysis

Log transformation was used for BUN, SCr, eGFR, TG, and HDL-C levels when their results were not normally distributed. Data are presented as mean ± SD for continuous variables, median (25, 75) for skewed variables, and percentages for categorical variables. We used the one-way ANOVA test to compare continuous characteristics, and the chi-squared test or Fisher exact test to compare percentages among groups. The correlations between renal function and independent variables were examined by Spearman’s correlation coefficient. Multivariable logistic analyses were performed to evaluate the association between eGFR and the presence of osteoporosis in different models. Two models were applied as unadjusted model and adjusted model which adjusted for age, menopausal duration, and BMI. All data were analyzed using SPSS version 13.0 (SPSS Inc., Chicago, IL, USA). *P* value less than 0.05 was considered statistically significant.

## Results

The characteristics of the study participants are presented in Table 1. A total of 776 relatively healthy postmenopausal Chinese women participated in the present study. The mean age was 62.1 ± 6.1 years. Osteoporosis in at least one site (lumbar spine, femoral neck or total hip) was found in 37.2% of participants. We compared various indices between subjects with and without osteoporosis. As shown in Table 1, participants with normal BMD were younger and had higher BMI and shorter menopausal duration (*P* < 0.001).

**Table 1:** Characteristics of participants according to BMD

Variables	Total (*n* = 776)	Normal (*n* = 487)	Osteoporosis (*n* = 289)	*P*
Age (years)	62.1 ± 6.1	61.0 ± 5.7	63.9 ± 6.2	< 0.001
Menopausal duration (years)	12.6 ± 7.1	11.1 ± 6.7	14.9 ± 7.3	< 0.001
Height (cm)	153.8 ± 5.1	154.5 ± 5.0	152.7 ± 5.2	< 0.001
Weight (kg)	55.4 ± 7.8	57.0 ± 7.6	52.7 ± 7.4	< 0.001
BMI (kg/cm^2^)	23.4 ± 3.0	23.9 ± 2.9	22.6 ± 2.9	< 0.001
BUN (mmol/l)	5.3 (4.4, 6.2)	5.3 (4.4, 6.3)	5.2 (4.4, 6.1)	0.455
SCr (μmol/l)	58.3 (49.4, 66.1)	58.7 (50.4, 65.4)	57.7 (47.2, 67.1)	0.316
eGFR (ml/min/ 1.73m^2^)^a^	94.7 (85.3, 101.5)	95.4 (86.6, 101.5)	93.5 (82.4, 101.8)	0.216
L1-L4 BMD (g/cm^2^)	0.933 ± 0.149	1.004 ± 0.121	0.814 ± 0.110	< 0.001
Femoral neck BMD (g/cm^2^)	0.735 ± 0.119	0.794 ± 0.098	0.634 ± 0.078	< 0.001
Total hip BMD (g/cm^2^)	0.814 ± 0.125	0.877 ± 0.098	0.707 ± 0.087	< 0.001
TG (mmol/l)	1.4 (1.1, 2.0)	1.5 (1.1, 2.0)	1.4 (1.1, 2.0)	0.342
TC (mmol/l)	5.3 ± 0.9	5.3 ± 0.9	5.3 ± 0.9	0.442
HDL-C (mmol/l)	1.5 (1.3, 1.8)	1.5 (1.3, 1.7)	1.6 (1.4, 1.8)	0.044
LDL-C (mmol/l)	3.1 ± 0.7	3.1 ± 0.7	3.1 ± 0.7	0.444
SCa (mmol/l)	2.4 ± 0.1	2.4 ± 0.1	2.4 ± 0.1	0.140
Cigarette smoking, %	13 (1.7%)	7 (1.4%)	6 (2.1%)	0.567
Alcohol consumption, %	20 (2.6%)	16 (3.3%)	4 (1.4%)	0.106
Physical exercise, %	639 (82.3%)	405 (83.2%)	234 (81.0%)	0.439

Data are expressed as mean ± SD, median (25, 75) or number with percentage (%) in parentheses

*BMD* bone mineral density, *BMI* body mass index, *BUN* blood urea nitrogen, *eGFR* estimated glomerular filtration rate, *HDL-C* high-density lipoprotein cholesterol, *LDL-C* low-density lipoprotein cholesterol, *SCa* serum calcium, *SCr* serum creatinine, *TC* total cholesterol, *TG* triglyceride

^a^eGFR calculated using the CKD Epidemiology Collaboration 2009 equation

The analysis according to renal function (Table 2) showed that participants with eGFR ≥90 ml/min/1.73m^2^ were younger and had shorter menopausal duration (*P* < 0.001). BMD values at femoral neck and total hip, but not at lumbar spine, were significantly lower in women with declining renal function (*P* < 0.01). As renal function decreased, the percentage of participants with osteoporosis increased (*P* < 0.05). As shown in Table 3, eGFR was negatively and significantly correlated with age and menopausal duration (*P* < 0.01). The levels correlated with femoral neck BMD and total hip BMD (*r* = 0.086, and *r* = 0.071, *P* < 0.05, respectively). SCr levels correlated with age (*r* = 0.107, *P* < 0.01), menopausal duration (*r* = 0.086, *P* < 0.05), and TC levels (*r* = 0.074, *P* < 0.05). BUN was positively correlated with age, menopausal duration (*P* < 0.01), TC, LDL-C, and SCa levels (*P* < 0.05). eGFR is computed according to age and SCr concentration. BMD could be influenced by age and menopausal duration. Therefore, we performed subgroup analysis stratified by age and menopausal duration. eGFR did not correlate with BMD at all sites after stratification by age or menopausal duration (Additional file 1: Tables S1 and S2).

**Table 2:** Characteristics of participants according to renal function, measured by eGFR

Variables	Category of eGFR^a^ (ml/min/1.73m^2^)		*p*-value
	< 90 (*n* = 273)	≥90 (*n* = 503)	
Age (years)	65.1 ± 6.0	60.4 ± 5.5	< 0.001
Menopausal duration (years)	15.4 ± 7.0	11.0 ± 6.7	< 0.001
Height (cm)	153.8 ± 4.9	153.8 ± 5.3	0.858
Weight (kg)	55.3 ± 7.3	55.5 ± 8.1	0.914
BMI (kg/cm^2^)	23.4 ± 2.8	23.4 ± 3.1	0.965
BUN (mmol/l)	5.7 (4.8, 6.6)	5.0 (4.2, 5.9)	< 0.001
SCr (μmol/l)	69.1 (65.0, 73.7)	53.3 (44.0, 58.6)	< 0.001
eGFR (ml/min/1.73m^2^)	80.3 (74.1, 86.2)	99.3 (95.3, 104.9)	< 0.001
L1-L4 BMD (g/cm^2^)	0.924 ± 0.142	0.938 ± 0.152	0.195
Femoral neck BMD (g/cm^2^)	0.717 ± 0.106	0.744 ± 0.125	0.004
Total hip BMD (g/cm^2^)	0.796 ± 0.116	0.823 ± 0.129	0.004
TG (mmol/l)	1.5 (1.1, 2.1)	1.4 (1.1, 2.0)	0.991
TC (mmol/l)	5.4 ± 0.9	5.3 ± 0.9	0.169
HDL-C (mmol/l)	1.5 (1.3, 1.7)	1.6 (1.3, 1.8)	0.350
LDL-C (mmol/l)	3.2 ± 0.7	3.1 ± 0.7	0.287
SCa (mmol/l)	2.4 ± 0.1	2.4 ± 0.1	0.849
Cigarette smoking, %	8 (2.9%)	5 (1.0%)	0.074
Alcohol consumption, %	6 (2.2%)	14 (2.8%)	0.623
Physical exercise, %	231 (84.6%)	408 (81.1%)	0.222
Osteoporosis,%	115 (42.1%)	174 (34.6%)	0.038

Data are expressed as mean ± SD, median (25, 75) or number with percentage (%) in parentheses

*BMD* bone mineral density, *BMI* body mass index; BUN, blood urea nitrogen, *eGFR* estimated glomerular filtration rate, *HDL-C* high-density lipoprotein cholesterol, *LDL-C* low-density lipoprotein cholesterol, *SCa* serum calcium, *SCr* serum creatinine, *TC* total cholesterol, *TG* triglyceride

^a^eGFR calculated using the CKD Epidemiology Collaboration 2009 equation

**Table 3:** Correlation analysis of renal function indicators and other variables

Variables	eGFR^c^	BUN	SCr
Age (years)	−0.426^a^	0.136^a^	0.107^a^
Menopausal duration (years)	−0.360^a^	0.133^a^	0.086^b^
BMI (kg/cm^2^)	−0.012	0.014	0.010
L1-L4 BMD (g/cm^2^)	0.057	0.014	0.022
Femoral neck BMD (g/cm^2^)	0.086^b^	−0.025	0.027
Total hip BMD (g/cm^2^)	0.071^b^	−0.022	0.016
TG (mmol/l)	0.018	−0.064	−0.022
TC (mmol/l)	−0.045	0.084^b^	0.074^b^
HDL-C (mmol/l)	0.038	0.061	−0.052
LDL-C (mmol/l)	−0.005	0.079^b^	0.025
SCa (mmol/l)	0.000	0.075^b^	0.028

*BMD* bone mineral density, *BMI* body mass index, *BUN* blood urea nitrogen, *eGFR* estimated glomerular filtration rate, *HDL-C* high-density lipoprotein cholesterol, *LDL-C* low-density lipoprotein cholesterol, *SCa* serum calcium, *SCr* serum creatinine, *TC* total cholesterol, *TG* triglyceride

^a^ *P* < 0.01

^b^ *P* < 0.05

^c^eGFR calculated using the CKD Epidemiology Collaboration 2009 equation. r, correlation coefficient

Table 4 shows the results of binary logistic regression analyses using osteoporosis as the dependent variable and eGFR groups as the independent variables. In the unadjusted model, lower eGFR class was associated with an increased risk of osteoporosis as compared to the higher eGFR class (odds ratio (OR): 1.38, 95% confidential interval (CI): 1.02–1.86, *P* < 0.05). However, after adjustment for age, menopausal duration, and BMI, the lower eGFR class was not related to an increase risk of osteoporosis as compared to the higher eGFR class (OR: 0.94, 95%CI: 0.67–1.33, *P* > 0.05). Subgroup analysis by age and menopausal duration also showed that the decline in renal function was not associated with osteoporosis risk (Additional file 1: Tables S3 and S4).

**Table 4:** Logistic regression of osteoporosis

	Unadjusted			Adjusted^a^		
	*P* value	OR	95% CI	*P* value	OR	95% CI
eGFR (ml/min/1.73m^2^)^b^						
≥90	0.039	1.00 (ref)		0.738	1.00 (ref)	
< 90		1.38	1.02–1.86		0.94	0.67–1.33

*CI* confidence interval, *eGFR* estimated glomerular filtration rate, *OR* odds ratio

^a^Adjustment for age, menopausal duration and BMI

^b^eGFR calculated using the CKD Epidemiology Collaboration 2009 equation

## Discussion

Several studies have concerned to evaluate the association between renal function and BMD [5–9, 13, 14]. However, the literatures are conflicting, and the relationship between renal function and the risk of osteoporosis in healthy older population is rare. In the current study, we examined the relationship of renal function with BMD and the risk of osteoporosis in 776 relatively healthy postmenopausal Chinese women. Our present study found that participants with worse renal function were associated with lower femoral neck and total hip BMD. However, after adjusting for age, menopausal duration and BMI, the decline in renal function was not associated with increased risk of osteoporosis as compared with normal renal function.

As for the relationship between renal function and BMD in the general population, the results are controversial. Kaji et al. [7] reported a positive relationship between eGFR, calculated using the Modification of Diet in Renal Disease (MDRD) equation for assessing renal function, and BMD in postmenopausal women. Similarly, a retrospective study of 1172 CKD outpatients also described an association between reduced BMD and impaired renal function (using the CKD-EPI equation) [5]. Ensrud et al. [26] suggested that lower eGFR, calculated using CKD-EPI 2012 equation, was associated with increased risk of hip fracture in older community-dwelling men. However, Hsu et al. [27] demonstrated that a decline in renal function was not associated with decreased BMD. Malmgren et al. [28] also showed that the prevalence of osteoporosis did not differ with renal function. In our study, although Spearman’s correlation analysis showed a positive relationship between eGFR and BMD values, this relationship was attenuated after adjustment for potential confounders. We suggest that the discrepancies might be caused by differences in different research population studied, the equations used, skeletal sites observed, and the higher rate of normal population in subjects of the present study. With MDRD equations, the eGFR tends to be underestimated in people with normal renal function [29]. CKD-EPI equations are shown to be more accurate than MDRD equations to assess renal function in older population and healthy individuals [30, 31].

In a study of 1,815,943 participants, the association was negative for eGFR and rates of fracture, but after adjusting for additional demographic variables and comorbidities, the relationship disappeared [13]. Similar to their findings, our study showed that decreased eGFR was associated with reduced BMD; however, after adjustment for age, menopausal duration and BMI, we found no evidence to support the hypothesis that the decline in renal function is independently associated with osteoporosis risk. Why was reduced eGFR not associated with increased osteoporosis risk, in contrast to the excess osteoporosis risk observed in patients with end-stage renal disease (ESRD) [32]? In comparison to earlier study, we focused on relatively healthy postmenopausal Chinese women, so we excluded subjects with impaired renal function (eGFR < 60 ml/min/1.73m^2^). This may explain in part why our results differ from previous studies [5], which included 415 CKD outpatients with eGFR < 60 ml/min/1.73^2^. In addition, we found that eGFR was associated with age and menopausal duration, both of which are known risk factors for osteoporosis [33, 34]. Thus, we assume that the association between eGFR and BMD can be explained by known confounding factors, such as age and menopausal duration.

Spearman’s correlation analysis of the data showed that SCr was not associated with BMD values. Since SCr levels are affected by muscle mass, it is not accurate for assessing renal function [35, 36]. Similar to our findings, Han et al. [8] did not find association between SCr and BMD.

Our study indicates that there are skeletal-site-specific differences in the relationships between BMD levels and eGFR. However, after stratification by age, eGFR did not correlate with BMD at all sites. Those with eGFR < 90 ml/min/1.73m^2^ were older compared with those with eGFR ≥90 ml/min/1.73m^2^. These findings indicate that the association of lower BMD with lower eGFR was due in large part to age. Several reasons could partly explain the skeletal site-specificity differences. First, the overlying aortic calcifications make it difficult to measure BMD at lumbar spine in the elderly [37]. Besides, present BMD does not reflect present bone metabolism alone, but rather integrates bone metabolism from the past to present. Finally, spine BMD remains approximately stable or increases over time, whereas BMD of the total hip and femoral neck declines at an increasing rate in elderly people [38, 39]. In the present study, we focus on relatively healthy subjects with eGFR ≥60 ml/min/1.73m^2^. Thus, further studies are needed to explore the relationship between reduced renal function and BMD in CKD.

The present study has notable strengths including its relatively large sample size, focus on the healthy postmenopausal Chinese women, where data on this population are rare. No participant had taken a drug known to affect bone metabolism and renal function. Besides, important confounding factors were adjusted in the regression analysis. The present study has several potential limitations. Firstly, due to the observational study design, it is impossible to establish a causal relation between renal function and BMD. A longitudinal follow-up study is necessary to ascertain these relationships. Another limitation, which should be mentioned, is that we excluded subjects with impaired renal function (eGFR < 60 ml/min/1.73m^2^). Thus, our findings might not be applicable to the entire population of Chinese postmenopausal women. In addition, this study included subjects with CKD stage 2 (defined an eGFR 60–90 ml/min/1.73m^2^) [23]. Thirdly, the great majority of studies concluded that formulas based on serum cystatin C are superior to SCr-based eGFR [26, 40]. However, Keddis et al. [41] showed that SCr-based CKD-EPI equation was preferred over cystatin C-based eGFR in kidney transplant recipients because they are less biased, more accurate. Moreover, the gold standard for detecting eGFR is inulin clearance, which is rarely done due to issues with inconvenience and time consuming for epidemiologic studies. Finally, urine protein or imaging examination, for example, is lacking for confirming impaired renal function.

## Conclusions

In conclusion, our data showed that although participants with declined eGFR have significantly reduced femoral neck and total hip BMD, this association disappeared after adjusting for age, menopausal duration and BMI. Renal function itself is not independently associated with the risk of osteoporosis in relatively healthy postmenopausal Chinese women.

## Supplementary information

**Additional file 1: Table S1.** Correlation analysis of renal function indicators and BMD (stratified by age). **Table S2.** Correlation analysis of renal function indicators and BMD (stratified by menopausal duration). **Table S3.** Logistic regression of osteoporosis (stratified by age). **Table S4.** Logistic regression of osteoporosis (stratified by menopausal duration).

## Data Availability

The datasets used and analysed during the current study are available from the corresponding author on reasonable request, once the study has been published.

## Abbreviations

BMD: Bone mineral density
BMI: Body mass index
BUN: Blood urea nitrogen
CI: Confidential interval
CKD: Chronic kidney disease
CKD-EPI: Chronic Kidney Disease Epidemiology Collaboration
DXA: Dual-energy X-ray absorptiometry
eGFR: Estimated glomerular filtration rate
ESRD: End-stage renal disease
HDL-C: High-density lipoprotein cholesterol
LDL-C: Low-density lipoprotein cholesterol
MDRD: Modification of Diet in Renal Disease
OR: Odds ratio
SCa: Serum calcium
SCr: Serum creatinine
SDs: Standard deviations
TC: Total cholesterol
TG: Triglyceride (TG)

## References

[1] Malmgren L, McGuigan FE, Berglundh S, Westman K, Christensson A, Åkesson K (2015). Declining estimated glomerular filtration rate and its association with mortality and comorbidity over 10 years in elderly women. Nephron.

[2] Nikolich-Žugich J, Goldman DP, Cohen PR, Cortese D, Fontana L, Kennedy BK, Mohler MJ, Olshansky SJ, Perls T, Perry D, Richardson A, Ritchie C, Wertheimer AM, Faragher RG, Fain MJ (2016). Preparing for an aging world: engaging biogerontologists, geriatricians, and the society. J Gerontol A Biol Sci Med Sci.

[3] Glassock RJ, Denic A, Rule AD (2017). The conundrums of chronic kidney disease and aging. J Nephrol.

[4] Coughlan T, Dockery F (2014). Osteoporosis and fracture risk in older people. Clin Med (Lond).

[5] Bezerra de Carvalho KS, Vasco RFV, Custodio MR, Jorgetti V, Moysés RMA, Elias RM (2019). Chronic kidney disease is associated with low BMD at the hip but not at the spine. Osteoporos Int.

[6] Chen H, Lips P, Vervloet MG, van Schoor NM, de Jongh RT (2018). Association of renal function with bone mineral density and fracture risk in the longitudinal aging study Amsterdam. Osteoporos Int.

[7] Kaji H, Yamauchi M, Yamaguchi T, Shigematsu T, Sugimoto T (2010). Mild renal dysfunction is a risk factor for a decrease in bone mineral density and vertebral fractures in Japanese postmenopausal women. J Clin Endocrinol Metab.

[8] Han W, Bai XJ, Han LL, Akhtari S, Sun XF, Chen XM (2018). Association between the age-related decline in renal function and lumbar spine bone mineral density in healthy Chinese postmenopausal women. Menopause.

[9] Kim HL, Park IY, Choi JM, Hwang SM, Kim HS, Lim JS, Kim M, Son MJ (2011). A decline in renal function is associated with loss of bone mass in Korean postmenopausal women with mild renal dysfunction. J Korean Med Sci.

[10] Jamal SA, Miller PD (2013). Secondary and tertiary hyperparathyroidism. J Clin Densitom.

[11] Miller PD (2014). Bone disease in CKD: a focus on osteoporosis diagnosis and management. Am J Kidney Dis.

[12] Hruska KA, Sugatani T, Agapova O, Fang Y (2017). The chronic kidney disease - mineral bone disorder (CKD-MBD): advances in pathophysiology. Bone.

[13] Elliott MJ, James MT, Quinn RR, Ravani P, Tonelli M, Palacios-Derflingher L, Tan Z, Manns BJ, Kline GA, Ronksley PE, Hemmelgarn BR (2013). Estimated GFR and fracture risk: a population-based study. Clin J Am Soc Nephrol.

[14] Fujita Y, Iki M, Tamaki J, Kouda K, Yura A, Kadowaki E, Sato Y, Moon JS, Tomioka K, Okamoto N, Kurumatani N (2013). Renal function and bone mineral density in community-dwelling elderly Japanese men: the Fujiwara-kyo osteoporosis risk in men (FORMEN) study. Bone.

[15] Cipriani C, Pepe J, Bertoldo F, Bianchi G, Cantatore FP, Corrado A, Di Stefano M, Frediani B, Gatti D, Giustina A, Porcelli T, Isaia G, Rossini M, Nieddu L, Minisola S, Girasole G, Pedrazzoni M (2018). The epidemiology of osteoporosis in Italian postmenopausal women according to the National Bone Health Alliance (NBHA) diagnostic criteria: a multicenter cohort study. J Endocrinol Investig.

[16] Gnudi S, Sitta E, Fiumi N (2007). Relationship between body composition and bone mineral density in women with and without osteoporosis: relative contribution of lean and fat mass. J Bone Miner Metab.

[17] Zeng Qiang, Li Na, Wang Qianqian, Feng Jian, Sun Dongmao, Zhang Qiu, Huang Jiyuan, Wen Qingxiang, Hu Rong, Wang Liang, Ma Yuanzheng, Fu Xiaoxia, Dong Shengyong, Cheng Xiaoguang (2019). The Prevalence of Osteoporosis in China, a Nationwide, Multicenter DXA Survey. Journal of Bone and Mineral Research.

[18] Alejandro P, Constantinescu F (2018). A review of osteoporosis in the older adult: an update. Rheum Dis Clin N Am.

[19] Baccaro LF, Conde DM, Costa-Paiva L, Pinto-Neto AM (2015). The epidemiology and management of postmenopausal osteoporosis: a viewpoint from Brazil. Clin Interv Aging.

[20] Si L, Winzenberg TM, Jiang Q, Chen M, Palmer AJ (2015). Projection of osteoporosis-related fractures and costs in China: 2010-2050. Osteoporos Int.

[21] Aleksova J, Kurniawan S, Elder GJ (2018). The trabecular bone score is associated with bone mineral density, markers of bone turnover and prevalent fracture in patients with end stage kidney disease. Osteoporos Int.

[22] Li S, Guo H, Liu Y, Wu F, Zhang H, Zhang Z, Xie Z, Sheng Z, Liao E (2015). Relationships of serum lipid profiles and bone mineral density in postmenopausal Chinese women. Clin Endocrinol.

[23] Inker LA, Astor BC, Fox CH, Isakova T, Lash JP, Peralta CA, Kurella Tamura M, Feldman HI (2014). KDOQI US commentary on the 2012 KDIGO clinical practice guideline for the evaluation and management of CKD. Am J Kidney Dis.

[24] Kanis JA (2002). Diagnosis of osteoporosis and assessment of fracture risk. Lancet.

[25] Wu XP, Liao EY, Zhang H, Shan PF, Cao XZ, Liu SP (2004). Establishment of BMD reference plots and determination of peak BMD at multiple skeletal regions in mainland Chinese women and the diagnosis of osteoporosis. Osteoporos Int.

[26] Ensrud KE, Parimi N, Fink HA, Ishani A, Taylor BC, Steffes M, Cauley JA, Lewis CE (2014). Orwoll ES; Osteoporotic Fractures in Men Study Group. Estimated GFR and risk of hip fracture in older men: comparison of associations using cystatin C and creatinine. Am J Kidney Dis.

[27] Hsu CY, Cummings SR, McCulloch CE, Chertow GM (2002). Bone mineral density is not diminished by mild to moderate chronic renal insufficiency. Kidney Int.

[28] Malmgren L, McGuigan F, Christensson A, Akesson KE (2017). Reduced kidney function is associated with BMD, bone loss and markers of mineral homeostasis in older women: a 10-year longitudinal study. Osteoporos Int.

[29] Lin J, Knight EL, Hogan ML, Singh AK (2003). A comparison of prediction equations for estimating glomerular filtration rate in adults without kidney disease. J Am Soc Nephrol.

[30] Omuse G, Maina D, Mwangi J, Wambua C, Kanyua A, Kagotho E, Amayo A, Ojwang P, Erasmus R (2017). Comparison of equations for estimating glomerular filtration rate in screening for chronic kidney disease in asymptomatic black Africans: a cross sectional study. BMC Nephrol.

[31] Matsushita K, Mahmoodi BK, Woodward M, Emberson JR, Jafar TH, Jee SH, Polkinghorne KR, Shankar A, Smith DH, Tonelli M, Warnock DG, Wen CP, Coresh J, Gansevoort RT, Hemmelgarn BR (2012). Levey AS; Chronic Kidney Disease Prognosis Consortium. Comparison of risk prediction using the CKD-EPI equation and the MDRD study equation for estimated glomerular filtration rate. JAMA.

[32] Gupta R, Mohammed AM, Alenizi EK, Ben NA (2011). Bone mineral density in Kuwaiti patients with end-stage renal disease. Med Princ Pract.

[33] Fistarol M., Rezende C. R., Figueiredo Campos A. L., Kakehasi A. M., Geber S. (2019). Time since menopause, but not age, is associated with increased risk of osteoporosis. Climacteric.

[34] Nielsen BR, Linneberg A, Christensen K, Schwarz P (2015). Perceived age is associated with bone status in women aged 25-93 years. Age (Dordr).

[35] Raman M, Middleton RJ, Kalra PA, Green D (2017). Estimating renal function in old people: an in-depth review. Int Urol Nephrol.

[36] Alaje AK, Adedeji TA, Adedoyin AR, Idogun SE (2019). Creatinine and cystatin C-based evaluation of renal function among obese subjects in Benin City, Nigeria. Saudi J Kidney Dis Transpl.

[37] Simon SP, Fodor D, Muntean L, Poanta L, Cristea P, Rednic S (2014). Bone mineral density, vertebral fractures and body mass index in postmenopausal women with abdominal aortic calcification. Endocr Res.

[38] Lim Y, Jo K, Ha HS, Yim HW, Yoon KH, Lee WC, Son HY, Baek KH, Kang MI (2017). The prevalence of osteoporosis and the rate of bone loss in Korean adults: the Chungju metabolic disease cohort (CMC) study. Osteoporos Int.

[39] McNabb BL, Vittinghoff E, Schwartz AV, Eastell R, Bauer DC, Ensrud K, Rosenberg E, Santora A, Barrett-Connor E, Black DM (2013). BMD changes and predictors of increased bone loss in postmenopausal women after a 5-year course of alendronate. J Bone Miner Res.

[40] Alaini A, Malhotra D, Rondon-Berrios H, Argyropoulos CP, Khitan ZJ, Raj DSC, Rohrscheib M, Shapiro JI, Tzamaloukas AH (2017). Establishing the presence or absence of chronic kidney disease: uses and limitations of formulas estimating the glomerular filtration rate. World J Methodol.

[41] Keddis MT, Amer H, Voskoboev N, Kremers WK, Rule AD, Lieske JC (2016). Creatinine-based and Cystatin C-based GFR estimating equations and their non-GFR determinants in kidney transplant recipients. Clin J Am Soc Nephrol.

