# Supplementary material for: Association between renal function and bone mineral density in healthy postmenopausal Chinese women
Source: BMC Endocr Disord. 2019 Dec 26;19:146. doi: 10.1186/s12902-019-0476-y (PMC6933739; doi:10.1186/s12902-019-0476-y)
Supplement: Supplementary file 1 — Additional file 1: Table S1. Correlation analysis of renal function indicators and BMD (stratified by age). Table S2. Correlation analysis of renal function indicators and BMD (stratified by menopausal duration). Table S3. Logistic regression of osteoporosis (stratified by age). Table S4. Logistic regression of osteoporosis (stratified by menopausal duration). [file 12902_2019_476_MOESM1_ESM.docx]

Table S1. Correlation analysis of renal function indicators and BMD (stratified by age)

| Variables | eGFR^*^ | BUN | SCr |
| --- | --- | --- | --- |
| Age ≤ 60 years |  |  |  |
| L1-L4 BMD (g/cm^2^) | -0.071 | 0.007 | 0.088 |
| Femoral neck BMD (g/cm^2^) | -0.023 | -0.018 | 0.036 |
| Total hip BMD (g/cm^2^) | -0.025 | -0.009 | 0.030 |
| Age 60-65 years |  |  |  |
| L1-L4 BMD (g/cm^2^) | -0.018 | 0.041 | 0.015 |
| Femoral neck BMD (g/cm^2^) | -0.003 | -0.041 | 0.005 |
| Total hip BMD (g/cm^2^) | 0.007 | -0.021 | -0.003 |
| Age ≥ 65 years |  |  |  |
| L1-L4 BMD (g/cm^2^) | -0.052 | 0.144^a^ | 0.059 |
| Femoral neck BMD (g/cm^2^) | -0.072 | 0.106 | 0.090 |
| Total hip BMD (g/cm^2^) | -0.076 | 0.071 | 0.094 |

^*^eGFR calculated using the CKD Epidemiology Collaboration 2009 equation. r, correlation coefficient.

BMD, bone mineral density; BUN, blood urea nitrogen; eGFR, estimated glomerular filtration rate; SCr, serum creatinine.

^a^*P* < 0.05

Table S2. Correlation analysis of renal function indicators and BMD (stratified by menopausal duration)

| Variables | eGFR^*^ | BUN | SCr |
| --- | --- | --- | --- |
| Menopausal duration ≤ 10 years | | | |
| L1-L4 BMD (g/cm^2^) | -0.031 | 0.082 | 0.060 |
| Femoral neck BMD (g/cm^2^) | -0.021 | 0.064 | 0.045 |
| Total hip BMD (g/cm^2^) | -0.014 | 0.068 | 0.024 |
| Menopausal duration 10-20 years | | | |
| L1-L4 BMD (g/cm^2^) | -0.004 | 0.007 | 0.008 |
| Femoral neck BMD (g/cm^2^) | 0.061 | -0.099 | -0.042 |
| Total hip BMD (g/cm^2^) | 0.038 | -0.070 | -0.025 |
| Menopausal duration ≥ 20 years | | | |
| L1-L4 BMD (g/cm^2^) | -0.155 | 0.139 | 0.134 |
| Femoral neck BMD (g/cm^2^) | -0.159 | 0.178^a^ | 0.178^*^ |
| Total hip BMD (g/cm^2^) | -0.159 | 0.094 | 0.161 |

^*^eGFR calculated using the CKD Epidemiology Collaboration 2009 equation. r, correlation coefficient.

BMD, bone mineral density; BUN, blood urea nitrogen; eGFR, estimated glomerular filtration rate; SCr, serum creatinine.

^a^*P* < 0.05

Table S3. Logistic regression of osteoporosis (stratified by age)

|  | Unadjusted | | | Adjusted^#^ | | |
| --- | --- | --- | --- | --- | --- | --- |
|  | P value | OR | 95%CI | P value | OR | 95%CI |
| Age ≤ 60 years | | | | | | |
| eGFR ≥90 | 0.532 | 1.00 (ref) |  | 0.474 | 1.00 (ref) |  |
| eGFR <90 |  | 0.81 | 0.42-1.56 |  | 0.78 | 0.40-1.53 |
| Age 60-65 years | | | | | | |
| eGFR ≥90 | 0.843 | 1.00 (ref) |  | 0.851 | 1.00 (ref) |  |
| eGFR <90 |  | 1.06 | 0.57-1.98 |  | 1.06 | 0.55-2.05 |
| Age ≥ 65 years | | | | | | |
| eGFR ≥90 | 0.980 | 1.00 (ref) |  | 0.812 | 1.00 (ref) |  |
| eGFR <90 |  | 1.01 | 0.61-1.65 |  | 0.94 | 0.56-1.58 |

eGFR calculated using the CKD Epidemiology Collaboration 2009 equation.

CI, confidence interval; eGFR, estimated glomerular filtration rate, ml/min/1.73m^2^; OR, odds ratio.

^#^Adjustment for age, menopausal duration and BMI.

Table S4. Logistic regression of osteoporosis (stratified by menopausal duration)

|  | Unadjusted | | | Adjusted^#^ | | |
| --- | --- | --- | --- | --- | --- | --- |
|  | P value | OR | 95%CI | P value | OR | 95%CI |
| Menopausal duration ≤ 10 years | | | | | | |
| eGFR ≥90 | 0.746 | 1.00 (ref) |  | 0.566 | 1.00 (ref) |  |
| eGFR <90 |  | 0.90 | 0.49-1.66 |  | 0.83 | 0.43-1.58 |
| Menopausal duration 10-20 years | | | | | | |
| eGFR ≥90 | 0.226 | 1.00 (ref) |  | 0.557 | 1.00 (ref) |  |
| eGFR <90 |  | 1.34 | 0.83-2.15 |  | 1.16 | 0.70-1.94 |
| Menopausal duration ≥ 20 years | | | | | | |
| eGFR ≥90 | 0.347 | 1.00 (ref) |  | 0.439 | 1.00 (ref) |  |
| eGFR <90 |  | 0.73 | 0.37-1.41 |  | 0.76 | 0.38-1.52 |

eGFR calculated using the CKD Epidemiology Collaboration 2009 equation.

CI, confidence interval; eGFR, estimated glomerular filtration rate, ml/min/1.73m^2^; OR, odds ratio.

^#^Adjustment for age, menopausal duration and BMI.
